# Protective efficacy of a Zika purified inactivated virus vaccine candidate during pregnancy in marmosets

**DOI:** 10.1038/s41541-024-00824-0

**Published:** 2024-02-17

**Authors:** In-Jeong Kim, Olga Gonzalez, Michael P. Tighe, Paula A. Lanthier, Madeline J. Clark, Kelsey L. Travis, Timothy C. Low-Beer, Kathleen G. Lanzer, Derek T. Bernacki, Frank M. Szaba, Rafael A. De La Barrera, Vincent Dussupt, Letzibeth Mendez-Rivera, Shelly J. Krebs, Corinna N. Ross, Stephanie D. Mdaki, Kathleen M. Brasky, Donna Layne-Colon, Suzette D. Tardif, Stephen J. Thomas, Kayvon Modjarrad, Marcia A. Blackman, Jean L. Patterson

**Affiliations:** 1 Trudeau Institute, Inc., Saranac Lake, NY 12983 USA; 2 Southwest National Primate Center, Texas Biomedical Research Institute, San Antonio, TX 78227 USA; 3 Pilot Bioproduction Facility, Center for Enabling Capabilities, Walter Reed Army Institute of Research, Silver Spring, MD 20910 USA; 4 Emerging Infectious Diseases Branch, Walter Reed Army Institute of Research, Silver Spring, MD 20910 USA; 5 U.S. Military HIV Research Program, Center of Infectious Disease Research, Walter Reed Army Institute of Research, Silver Spring, MD 20910 USA; 6 Henry M. Jackson Foundation for the Advancement of Military Medicine, Bethesda, MD 20817 USA; 7 Institute for Global Health and Translational Sciences, State University of New York, Upstate Medical University, Syracuse, NY 13210 USA; 8 Present Address: Science and Technology, Joint Base San Antonio-Fort Sam AFB, San Antonio, TX 78236 USA; 9 Present Address: Pfizer Inc. Vaccine Research and Development, Pearl River, NY 10965 USA

**Keywords:** Inactivated vaccines, Viral infection

## Abstract

Zika virus (ZIKV) infection during pregnancy poses significant threats to maternal and fetal health, leading to intrauterine fetal demise and severe developmental malformations that constitute congenital Zika syndrome (CZS). As such, the development of a safe and effective ZIKV vaccine is a critical public health priority. However, the safety and efficacy of such a vaccine during pregnancy remain uncertain. Historically, the conduct of clinical trials in pregnant women has been challenging. Therefore, clinically relevant animal pregnancy models are in high demand for testing vaccine efficacy. We previously reported that a marmoset pregnancy model of ZIKV infection consistently demonstrated vertical transmission from mother to fetus during pregnancy. Using this marmoset model, we also showed that vertical transmission could be prevented by pre-pregnancy vaccination with Zika purified inactivated virus (ZPIV) vaccine. Here, we further examined the efficacy of ZPIV vaccination during pregnancy. Vaccination during pregnancy elicited virus neutralizing antibody responses that were comparable to those elicited by pre-pregnancy vaccination. Vaccination also reduced placental pathology, viral burden and vertical transmission of ZIKV during pregnancy, without causing adverse effects. These results provide key insights into the safety and efficacy of ZPIV vaccination during pregnancy and demonstrate positive effects of vaccination on the reduction of ZIKV infection, an important advance in preparedness for future ZIKV outbreaks.

## Introduction

Zika virus (ZIKV) is an arbovirus transmitted by *Aedes* mosquito species. The 2015 ZIKV outbreak in Brazil revealed teratogenic features of ZIKV. Infection during pregnancy was shown to result in miscarriages and congenital Zika syndrome (CZS) in newborns, characterized by abnormal brain development and neurologic complications^1–5^. These devastating clinical outcomes prompted the World Health Organization to declare the ZIKV epidemic a public health emergency of international concern in 2016^6,7^. Approximately 20–30% of exposures to ZIKV during pregnancy resulted in health complications^8^. Approximately 5–14% of newborns were afflicted with abnormal development of the central nervous system (CNS), including microcephaly^9^. In addition, some newborns who were phenotypically normal at birth developed health problems as they grew older, including poor muscle control and impaired hearing and vision^8,10–13^. A fast track Zika vaccine development effort launched in 2016 resulted in the development of more than 50 ZIKV vaccine candidates^14,15^. Among these, a handful of vaccine candidates completed at least phase 1 clinical trials, which were reported to have favorable safety profiles and to be immunogenic in healthy individuals^16–19^. Pregnant women were largely excluded from clinical trials of ZIKV vaccines because of unknown potential risks to the developing fetus. Currently, no FDA-approved vaccines or therapeutics are available. Since 2018, the ZIKV epidemic has waned, presumably due to immunity from the last outbreak, making it almost impossible to conduct clinical end-point efficacy trials. However, sporadic ZIKV transmission continues, and poses a potential health threat to travelers^20,21^ with the potential for future regional epidemics.

WHO’s Zika virus Research and Development Roadmap has targeted the development of products for women of child bearing potential for emergency use in the next outbreak, rather than for preventive use^22^. This leaves pregnant women and women of child-bearing age at high risk of exposure to ZIKV and its clinical manifestations. Effective vaccines that target both infection and clinical disease, that are safe and effective when administered during pregnancy, will be in demand during future outbreaks. Therefore, research evaluating the ability of ZIKV vaccines to prevent maternal-fetal infections and CZS using relevant animal pregnancy models is critical.

ZIKV infection in non-human primates results in abnormalities in approximately 5–10% of infections, similar to that in humans^23–26^. A more consistent pattern has been shown for common marmosets, litter-bearing monkeys. We and others have shown that a two-dose intramuscular injection of the Brazilian SPH2015 strain of ZIKV (ZIKV-BR) 4 days apart consistently resulted in virus transmission from mothers to fetuses, or abortion within 16 days post infection (dpi)^27,28^. This consistency makes the marmoset pregnancy model ideal for testing vaccine candidates and therapeutics in preparation for future real-world Zika outbreaks. In addition, marmosets have the advantage of producing multiple offspring per pregnancy, significantly increasing the sample size for analyzing viral transfer to the fetus. Therefore, the marmoset pregnancy model offers unique benefits for understanding and combating ZIKV-associated congenital diseases.

Previously, a Zika purified inactivated virus (ZPIV) vaccine was shown to induce durable protective immunity in non-pregnant mice and macaques^29,30^ and exhibited a favorable safety and immunogenicity profile in humans^19,31,32^. Furthermore, we showed that pre-pregnancy vaccination of ZPIV prevented ZIKV-induced fetal demise in immunocompetent C57BL/6 mice and induced durable immunity in marmosets that was protective up to 72 weeks post vaccination against ZIKV challenge during pregnancy^28^. However, the safety and efficacy of ZPIV administered during pregnancy have not been evaluated in pregnant non-human primates. In this study, using the marmoset model, we show that the prime-boost vaccination (2.5 µg/dose) during pregnancy by the intramuscular route is well-tolerated without causing side effects, and elicits a robust neutralizing antibody response that may play a role in protection of the mother and the fetus against ZIKV infection. The results suggest that ZPIV is a promising vaccine candidate. These positive results support the importance of continuing investigation in the marmoset and other NHP models, and ultimate advancement to clinical research involving pregnant women, in preparation for future Zika outbreaks.

## Results

### Protection by ZPIV vaccination during pregnancy in marmosets

**Fig. 1:**
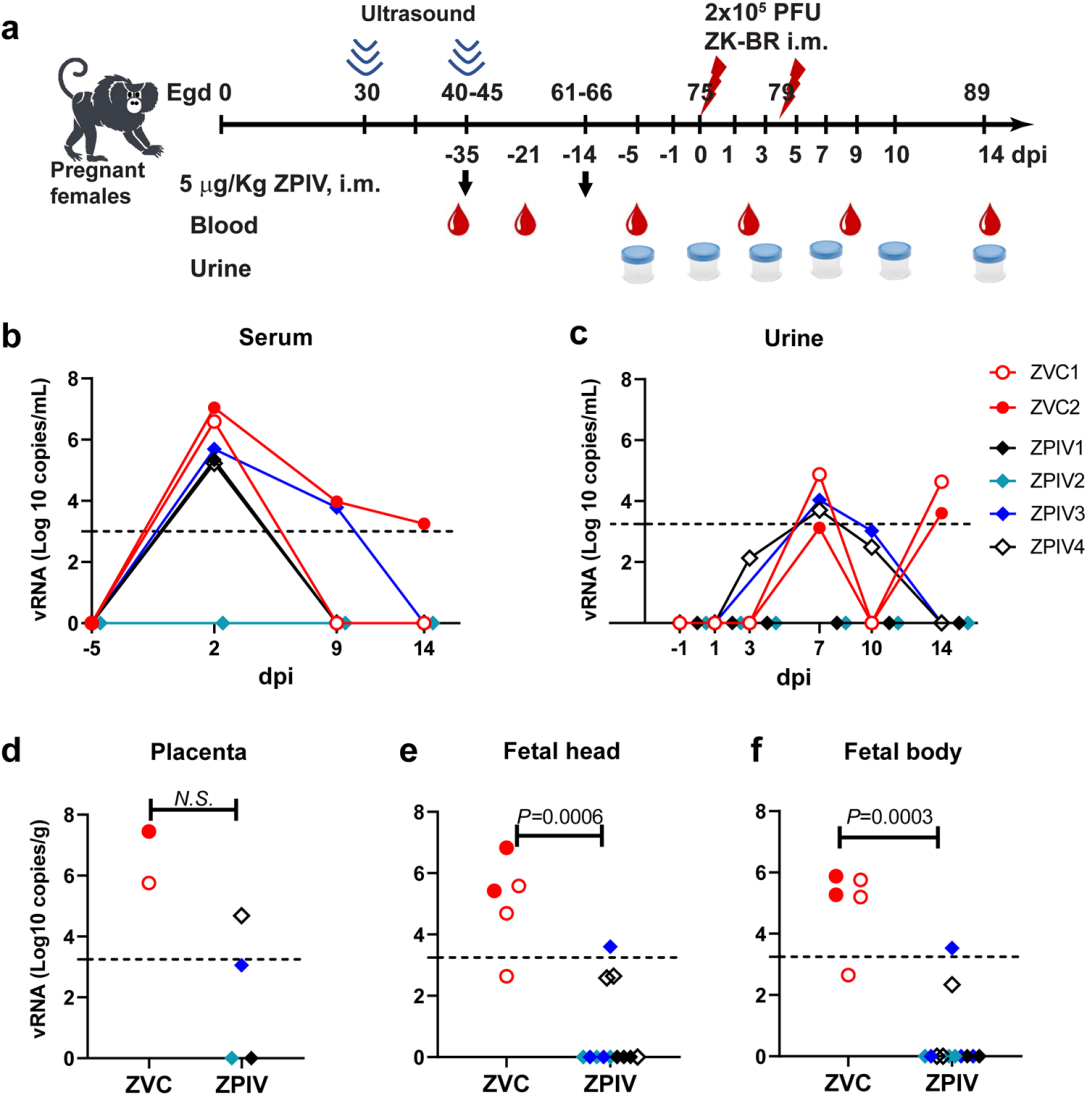
Protection by ZPIV vaccination during pregnancy in marmosets. Pregnant females (*n* = 4) received intramuscular injections of 2.5 μg alum adjuvanted ZPIV at egd 40 and 61. Then, 2 weeks after the boost, the marmosets received i.m. injections of 2.5 × 10^5^ PFU of ZIKV twice at egd 75 and 79. As controls, unvaccinated pregnant marmosets (*n* = 2, ZVC) were included and infected with the virus at comparable gestational days (egd 75 and 79) of pregnancy (**a**). All marmosets were bled at 0, 2, and 5- weeks post-prime vaccination as well as −5, 2, 9, and 14 dpi. Urine samples were prepared at −1, 1, 3, 7, 10 and 14 dpi. At 14 dpi (egd 89), dams were sacrificed, and the placentas and fetuses were extracted. Viral genomic RNA levels were quantitated using Real-Time RT-qPCR in total RNA prepared from serum (**b**), urine (**c**) at indicated time, placental tissues (**d**) at three locations per marmoset, a half of fetal head (**e**) and a half of fetal body (**f**). Dotted line indicates the limit of quantitation (Ct value < 35). Data were analyzed using the Mann–Whitney test to detect significant differences between groups. The ANOVA test was used to determine significant differences between group and time.

Taking advantage of the long gestational period (143–150 days) of marmosets, we examined whether ZPIV vaccination during pregnancy is safe and protective against ZIKV challenge during pregnancy. Marmosets received the first dose of 2.5 μg alum-adjuvanted ZPIV (5 μg kg^−1^ body weight) during the first trimester, at estimated gestational day (egd) 40, when pregnancy was confirmed (outlined in Fig. 1a). Marmosets received the second dose 3 weeks (egd 61–66) later. No obvious signs of distress, redness at the site of injection, shivering or limping were detected in the marmosets after vaccination when compared with an age-matched, gestational day comparable unvaccinated, virus-free control (VFC) dam, suggesting that ZPIV was well-tolerated.

Two weeks after the boost (egd 75–80), during the second trimester, marmosets were challenged with two-doses (2.5 × 10^5^ PFU/dose) of ZIKV-Brazil SPH2015 (ZK-BR) via the intramuscular route 4 days apart and euthanized at 14 dpi (egd 89–94). We have previously shown that the two-dose infection protocol produced consistent vertical transmission during pregnancy and maximized the potential for ZIKV-associated effects in pregnancy prior to abortion at 16 dpi^27^.

At 2 dpi, vRNA (10^6^–10^7^ copies mL^−1^) was detected in serum samples in two unvaccinated ZIKV infected control dams (ZVC 1 and 2). Approximately 100-fold lower vRNA copies (~10^5^ copies mL^−1^) were detected in 3 out of 4 ZPIV-vaccinated dams (ZPIV1, 3 and 4) while the other ZPIV-vaccinated dam, ZPIV2, showed no detectable vRNA at 2 dpi. Due to the variability in the vaccine group, the difference in viral RNA levels between the unvaccinated ZVC and vaccinated ZPIV groups was not significant (*P* = 0.133, Fig. 1b). However, substantial reduction in vRNA burden in the majority of vaccinated animals may suggest a vaccine effect. Due to the limited frequency and volume of blood collections, to avoid stress-associated negative impacts on pregnancy, we did not determine the precise kinetics of viral clearance at the RNA level. Viral RNA in the serum samples from all 4 ZPIV-vaccinated dams and one control dam became undetectable by 14 dpi, whereas vRNA in the unvaccinated ZVC2 was still above the limit of detection. In addition, at day 7, all marmosets had relatively comparable levels of vRNA in the urine. By day 14, the ZPIV group had cleared the virus from the urine whereas virus in the ZVC group recrudesced on day 14 (Fig. 1c). It is unclear whether the viral RNA detected in the serum and urine samples are translatable to infectious virus particles or remnant of sub-genomic viral RNA. Regardless of the possible presence of infectious virions, vaccination during pregnancy reduced viral burden and cleared virus within day 14 after ZIKV challenge.

We further assessed viral load in the placenta, fetal head and fetal body. In the ZVC group, vRNA (log10 copy number per gram tissue) was detected in all of the placentas (*n* = 2, the median of 6.6) and the fetuses (*n* = 5, levels ranging from a median of 5.4 and 5.27 in the fetal head and body, respectively), indicating viral transmission during pregnancy as expected. In the ZPIV group, two out of four placentas showed no detectable vRNA and the other two placentas showed detectable vRNA levels, substantially lower than those of the control, which were not significantly different due to the individual variability within the group (Fig. 1d). However, out of 12 fetuses from the vaccine group, 9 showed no detectable vRNA. In the cases where RNA was detected in fetal heads (*n* = 3) and bodies (*n* = 2), vRNA levels were significantly lower (*P* = 0.0006 and *P* = 0.0003, respectively) than in the ZVC group (Fig. 1e, f). These results indicate that ZPIV vaccination during pregnancy substantially reduces viral copy number in the placenta, which coincides with a significant reduction of vertical transmission into the fetus after infection during the late second trimester of pregnancy. We previously showed that infectious viral particles were not detectable in marmosets with vRNA copies lower than 10^5^ per gram tissue^28^. It is of interest to determine whether the presence of low levels (<10^4^ copies) of vRNA would have caused CZS in the fetuses in the vaccine group, however this was not studied here as we did not examine the post-birth effects.

### Prevention of ZIKV-caused placental pathology

**Fig. 2:**
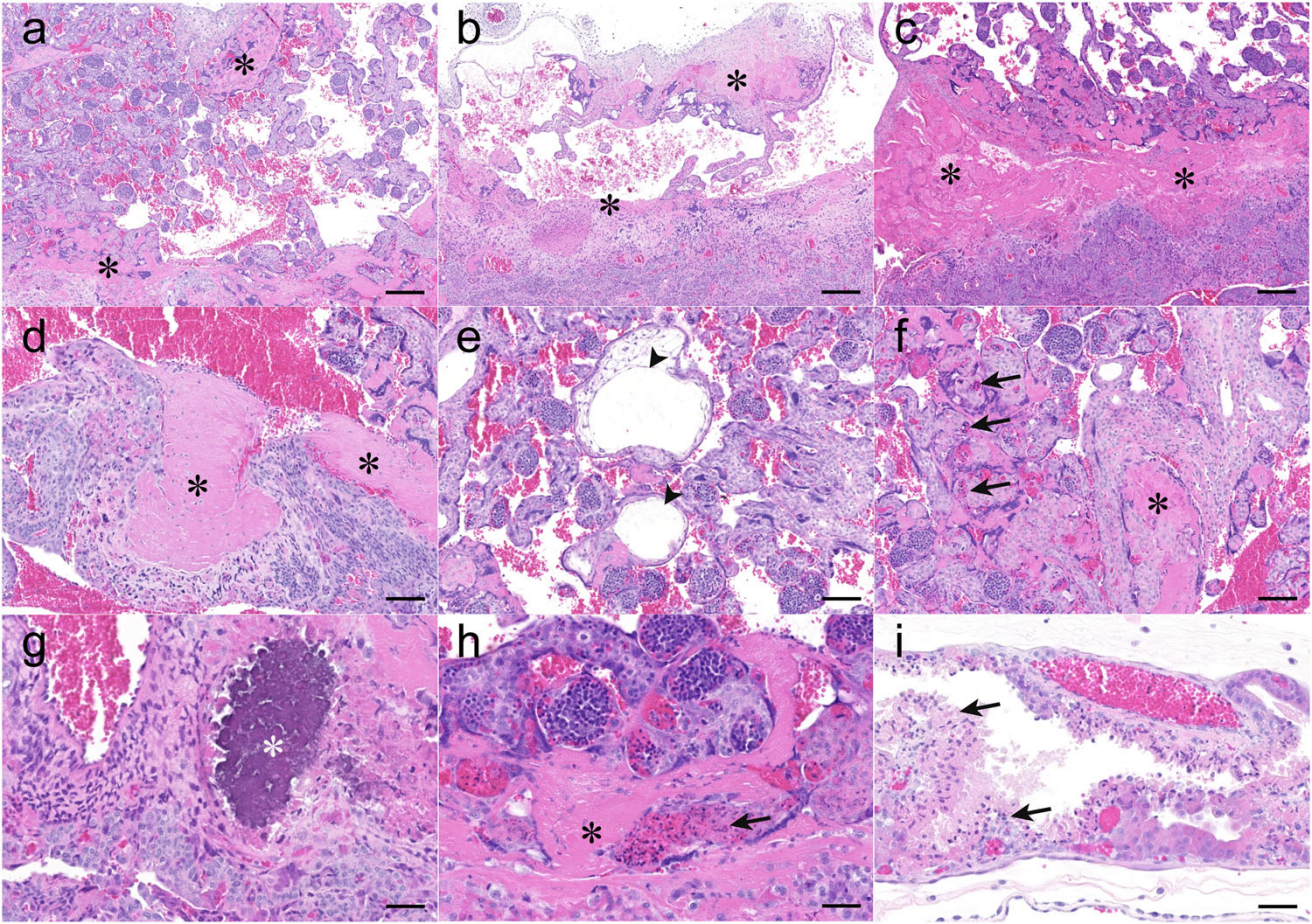
Placental pathology after ZIKV infection in unvaccinated marmosets. At 14 dpi, the placental images from the ZVC group present regional villous attenuation represented by decreased branching of villi and increased intervillous space flanked by fibrin deposits (**a**–**c**). Laminar layers of fibrin are consistent with thrombosis of maternal vasculature (**d**). Edema (arrowhead) resulted in villous expansion in the trabecular region (**e**). Extramedullary hematopoiesis necrosis (arrow) is found in the proximity of intervillous fibrin deposits (**f**). In a higher magnification (**g**–**i**), rare occasions of mineralization (white asterisk) are observed within decidualized stroma (**g**). Extramedullary hematopoiesis necrosis (arrow) is surrounded by intervillous fibrin deposits (**h**). Yolk sac epithelial necrosis (arrow) is extensive (**i**). Black asterisks indicate fibrin deposition throughout the placenta. Scale bars indicate 200 μm in (**a**–**c**), 100 μm in (**d**–**f**), and 50 μm in (**g**–**i**).

**Fig. 3:**
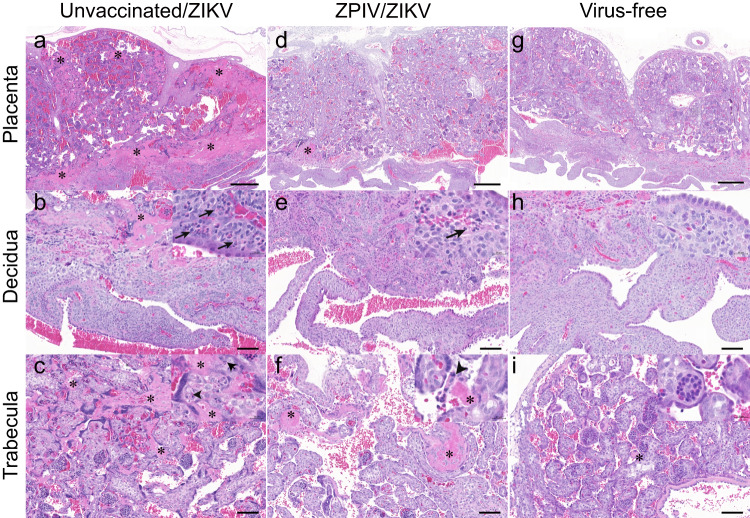
Reduction of ZIKV-induced inflammation and pathology in the placenta. At 14 dpi, the placentas at comparable gestational day ~89 were fixed and stained with hematoxylin and eosin. The representative images of the placentas of unvaccinated (**a**–**c**), vaccinated (**d**–**f**), and virus-free naive (**g**–**i**) dams. The top row represents the whole placenta, middle row represents decidua, and the bottom row represents trabecular (villous) areas. Decidua polymorphonuclear infiltration is indicated with an arrow. Asterisks indicate fibrin deposition. Arrow heads indicate necrotic bodies in the extramedullary hematopoiesis. Scale bars in (**a**), (**d**) and (**g**) are 500 μm. Sale bars in middle and bottom rows are 100 μm and 20 μm in the inserts.

ZIKV infection caused severe placental histopathology characterized by extensive fibrinoid depositions in the intervillous space as shown in unvaccinated ZVC marmosets (Fig. 2a–c), which contributes to regional villous attenuation and thrombosis of maternal vasculature (Fig. 2d). In addition, we observed inflated villi in the trabecular region due to edema (Fig. 2e), extramedullary hematopoiesis necrosis in the trabeculae (Fig. 2f), mineralization in the decidua region (Fig. 2g), and fibrin deposition associated extramedullary hematopoiesis necrosis (Fig. 2h) and yolk sac (Fig. 2i) in the placenta after ZIKV infection. At 14 dpi, the most prominent feature associated with ZIKV infection is fibrinoid deposition interspersed in the intervillous space (in a range of 11–43% of the placental mass among 9 placental blocks) of the ZVC group (Fig. 3a), which is high relative to that of the virus-free control (VFC) dam (Fig. 3). In humans, massive perivillous fibrin deposits (MPFD, higher than 25% of the placental mass) are associated with placental dysfunction, increasing the risk of intrauterine fetal growth restriction (IUGR)^33–35^, preterm delivery and neonatal mortality^36,37^, and impaired neurodevelopment^38^. In addition, signs of inflammation were evident in the unvaccinated ZVC dams, indicated by infiltration of polymorphonuclear cells in the decidua (Fig. 3b) and scattered necrotic bodies in the extramedullary hematopoiesis throughout the trabecular zone (Fig. 3c).

**Fig. 4:**
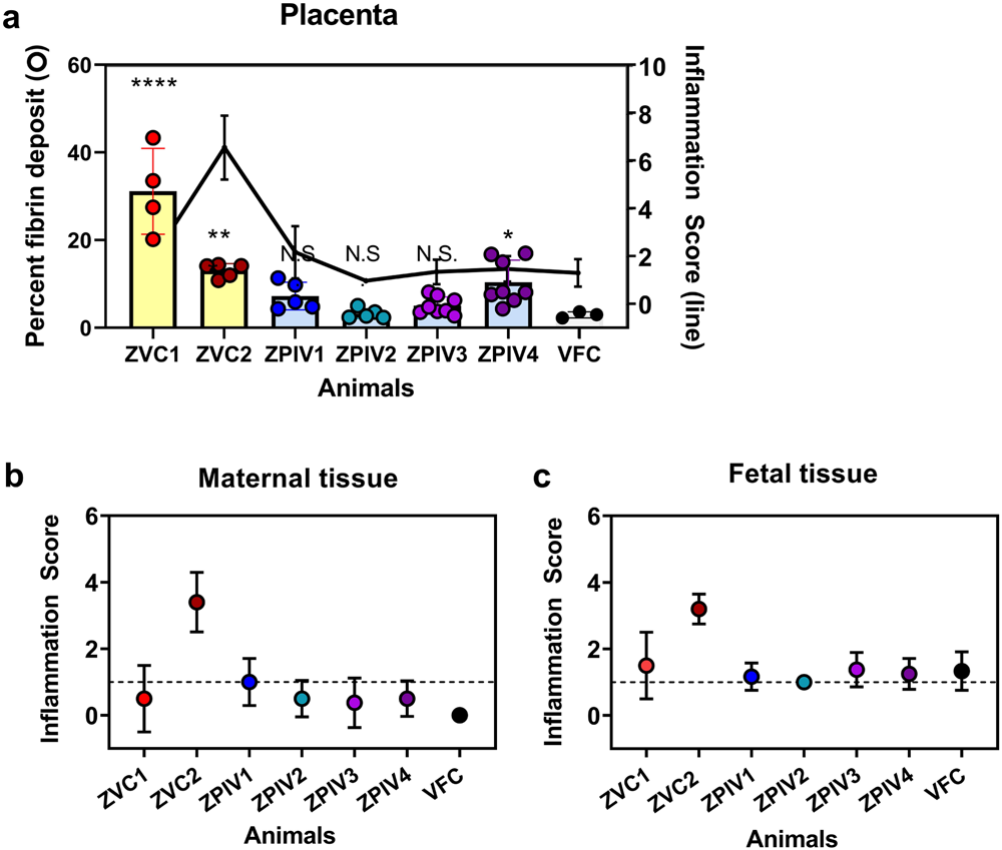
Fibrin depositions and inflammation scores in the placentas. At 14 dpi, multiple tissue blocks per placenta were stained with hematoxylin and eosin and examined. The areas of fibrin deposition were measured using HALO image analysis Platform software and presented as percent fibrin deposit (circles with bar) of the individual placentas (**a**). One-way ANOVA test was used to analyze the difference between virus-free control (VFC) and each of the individual animals. Asterisk indicates significant difference, ****P < 0.0001; ***P = 0.0024; *P = 0.0134; N.S., not significant (*P* > 0.05). A spectrum of inflammation in the placentas (black line) was assessed in a semi-quantitative method described in the materials and methods. Inflammation in the maternal tissue, decidua and intertrabecular space, (**b**) or fetal tissue, trabecular villi, (**c**) of the placentas are presented as the mean of 3–8 individual tissue blocks per placenta (±standard deviation). Dotted line represents mild inflammation score 1.

In the vaccinated group, fibrin depositions appeared to be less frequent and smaller in size (Fig. 3d). Moreover, polymorphonuclear cell infiltration in the decidua (Fig. 3e) and necrosis were less intense in the vaccinated dams (Fig. 3f), becoming more similar to the virus-free control dam (Fig. 3g–i). The comparative analyses of the placentas support that ZPIV vaccination during pregnancy reduced histopathology associated with ZIKV infection. Furthermore, we examined 5–8 tissue blocks per individual placenta, based on the semi-quantitative evaluation of inflammation, as described in the materials and methods. The overall magnitude of fibrin deposition appears to be independent of the severity of inflammation (Fig. 4a), supported by the ZVC group. ZVC1 showed prominent fibrin depositions (an average of 31.1% from 4 tissue locations) and a comparable degree of inflammation with the placenta of the virus-free control dam, whereas the ZVC2 placenta showed relatively low fibrin depositions (an average of 13.01% from 5 tissue locations) but intense inflammation. The spectrum of inflammation varied in the intervillous space, decidua (maternal tissue, Fig. 4b) and the trabecula villi (fetal tissue, Fig. 4c) among the individual placentas. In contrast, the magnitude of fibrinoid deposition and inflammation in the placenta of the ZPIV group was similar to that (an average of 2.93%) of the virus-free control, with the exception of ZPIV4 (an average of 10.39%), in which fibrinoid deposition was higher (*P* = 0.0134) than that of the virus-free control. The relatively high fibrinoid deposition in ZPIV4 might be associated with vRNA burden (4.8 × 10^4^ copies/g) in the placenta (Fig. 1d). Despite the individual variability, these data support the positive effect of ZPIV vaccination during pregnancy on the reduction of ZIKV-induced placental pathology.

### Robust neutralizing antibody response elicited by ZPIV vaccination during pregnancy

It is important to verify that vaccination during pregnancy elicits protective immunity. We examined virus neutralizing antibody titers using a microneutralization assay (Table 1), following the prime-boost ZPIV vaccination during the first trimester and early second trimester 3 weeks apart. At 4 weeks post prime (one-week post-boost), the geometric mean log MN_50_ titer (GMT) reached 2.77 (95% confident interval (C.I.) 2.47–3.07) above the threshold titers (log 10 MN_50_ titers ≥ 2.4) required for protection against ZIKV infection during pregnancy, as we reported previously^28^. These titers further increased to GMT 3.23 log 10 MN_50_ titer (95% C.I., 2.97–3.5) prior to ZIKV infection at 5 weeks post-prime. At 14 dpi, neutralizing antibody titers were almost 10-fold higher (GMT of 4.39) in the ZPIV group than the unvaccinated ZVC group (GMT of 3.5), coinciding with approximately a 100-fold reduction of viral RNA in the placentas and the significant reduction in the fetal heads and bodies (*P* = 0.0006 and *P* = 0.0003, respectively, as shown in Fig. 1) in the ZPIV group. These results suggest that ZPIV-elicited virus neutralizing antibodies reduce ZIKV infection during pregnancy. These are important findings because it was unclear whether pregnancy-associated biophysiological and hormonal changes would interfere with the immunogenicity and the potency of vaccination during pregnancy^39,40^. Specially, we found that virus-neutralizing antibody titers at 2 weeks post- the second dose immunization (boost) and day 9 post-ZIKV challenge in the vaccine group (ZPIV1-4) were comparable to those in the marmosets (V1-4) which were vaccinated before pregnancy in the previously published work^28^ (Supplementary Table 1). The current results demonstrate that ZPIV vaccination during pregnancy elicits a robust antibody response that contribute to the reduction of viral burden and virus-induced pathology in the placenta and fetal heads.

**Table 1:** Neutralizing antibody titers (Log10 MN_50_ titers) in marmosets after vaccination during pregnancy

Post-prime (week)	0	2	4	5	5.3	6.3	7
Est. gestation (day±3)	40	47	68	75	77	85	89
ZIKV challenge				0 dpi	2 dpi	9 dpi	14 dpi
ZVC1	0.70	0.70	0.70	0.70	0.70	1.56	3.27
ZVC2	0.70	0.70	0.70	0.70	0.70	3.30	3.72
GMT	0.70	0.70	0.70	0.70	0.70	2.44	3.50
Lower 95% C.I.	0.70	0.70	0.70	0.70	0.70	−8.66	0.65
Upper 95% C.I.	0.70	0.70	0.70	0.70	0.70	13.52	6.35
ZPIV1	0.70	2.11	2.69	3.27	3.53	3.87	3.86
ZPIV2	0.70	2.02	2.58	3.01	3.22	3.87	3.86
ZPIV3	0.70	0.70	3.02	3.42	3.58	4.36	4.73
ZPIV4	0.70	1.11	2.80	3.24	3.17	4.41	5.11
GMT	0.70	1.48	2.77	3.23	3.38	4.12	4.39
Lower 95% C.I.	0.70	0.39	2.47	2.97	3.04	3.65	3.39
Upper 95% C.I.	0.70	2.58	3.07	3.50	3.71	4.60	5.40
* p*-value		0.287	0.0001	<0.0001	<0.0001	0.0012	0.168

Marmosets were immunized with 2.5 μg alum adjuvanted ZPIV at 0 and 3 weeks or remain unimmunized. Then, all marmosets were bled at week 0 (prior to 1st vaccine), 2, 4 (1-week post-boost), and 5 (2-weeks post-boost) prior to ZIKV challenge, then, 2, 9, and 14 days after challenge. ZVC, unvaccinated, Zika virus infected control, ZPIV, zika purified inactivated virus, C.I., confident interval, GMT, geometric mean. Statistical difference between the ZVC and ZPIV groups was analyzed using ANOVA test. Within the unvaccinated ZVC group, MN_50_ titers at day 89 were significantly different (*P* = 0.0318) from the titers at day 77 but not from day 85 (*P* = 0.2426). Within the vaccinated ZPIV group, MN_50_ titers at day 77 were not significantly different (*P* > 0.39) from those at day 85 or day 89.

In an attempt to test the role of virus-neutralizing antibodies in vaccine-mediated protection, we examined the effect of passively transferring purified IgG from ZIKV hyperimmune humans, vaccine-derived IgG (vIgG), or purified IgG from normal human controls (cIgG). Two pregnant marmosets received a single dose of 25 mg vIgG (eq. 50 mg kg^−1^ body weight) and a single pregnant marmoset received two doses of 20 mg vIgG prior to ZIKV infection. In an independent mouse study, the dose equivalent to 37 mg kg^-1^ provided protection^41^. Previously, we reported that virus neutralizing antibody titers (log10 MN_50_ titers) higher than 2.4 provide protection against ZIKV challenge during pregnancy^28^. On days 0 and 2, neutralizing antibody titers in the recipients were 1.2–1.9. At 14 dpi, the current results showed no protection with the 25 mg dose, in terms of viral RNA in the lymph nodes and placenta but did show a reduction in viral load in the placenta with the two doses of 20 mg transfer (Supplementary Fig. 2). In addition, vRNA was close to or below the limit of detection in the fetal head and fetal body. The lack of protection provided by the single dose and further reduction of vRNA burden provided by the two-dose regimen suggests that the dosing and frequency of antibody treatment may need to be carefully optimized in future studies.

## Discussion

Safe and effective vaccines are urgently needed for preventing infection in pregnant mothers and fetuses. However, testing vaccine efficacy in pregnant women is highly restricted due to the theoretical risks of adverse effects. Using the marmoset pregnancy model in which ZIKV infection during pregnancy consistently results in vertical transmission from mother to fetus^27^, we report that ZPIV vaccination during pregnancy was well tolerated without vaccination-associated negative effects on pregnancy. ZPIV vaccination during pregnancy resulted in the reduction of placental pathology and a significant reduction of viral RNA burden in fetuses. These results underscore the benefits of ZPIV vaccination during pregnancy in the control of ZIKV infection.

In marmosets, ZIKV infection caused prominent fibrin depositions throughout the placentas at 14 dpi. In humans, massive perivillous fibrin deposition is associated with an increased risk of adverse pregnancy outcome such as intrauterine fetal growth restriction (IUGR)^34,35^, preterm birth^37^, and autoimmunity^42^. The excessive fibrin depositions in the marmoset placentas after ZIKV infection may be linked to previously described abortion at 16 dpi^27^. The impact of ZIKV infection on fetal development in pregnant marmosets offers a promising experimental NHP model for studying in-depth interactions between virus and fetal development, warranting further investigation.

Pregnant women experience profound immunologic changes for the maintenance of a successful pregnancy and the delivery of a semi-allogeneic fetus^43^. The immunogenicity and efficacy of vaccination in pregnant women have not been fully understood due to high risks of potentially harmful effects on fetus and mothers. A particular advantage of NHP pregnancy models of ZIKV is the relatively long gestational period, similar to humans, which allows us to test the immunogenicity of the vaccine during pregnancy. One of the key questions of this study was whether ZPIV vaccination during pregnancy elicits robust maternal immunity against ZIKV that protects the fetus. In our previous study^28^ that evaluated the efficacy of pre-pregnancy ZPIV vaccination, we observed that marmosets maintaining virus neutralizing-antibody titers higher than 2.4 log_10_ MN_50_ before challenge were able to protect their fetuses against in utero ZIKV infection. In the current study, ZPIV vaccination during pregnancy achieved log_10_ MN_50_ titers higher than 3 (GMT 3.23 with a range of 3.01–3.42). This exceeds the previously determined minimal virus neutralization antibody titer required for protecting against the identical ZIKV challenge condition. The current results show that ZPIV vaccination during pregnancy is highly immunogenic, eliciting a neutralizing antibody response that is comparable with the response of marmosets vaccinated prior to pregnancy (Supplementary Table 1)^28^. Although the outcomes of ZPIV vaccination during pregnancy were favorable with the reduction of placental pathology and reduction in vertical transmission, future investigations need to address (1) whether the immune response—especially the antibody response by vaccination during pregnancy—differs qualitatively from that of non-pregnant hosts, and (2) whether vaccination during pregnancy successfully prevents Zika-associated pregnancy loss, pre-mature births, and cognitive developmental abnormalities in infants.

Vaccination with inactivated virus (e.g., inactivated influenza virus) during pregnancy has been shown to be safe, with minimal reactogenicity and no negative interference with successful pregnancy outcomes for both mothers and fetuses^44,45^. Exploiting maternal immunity to prevent diseases in infants is routinely practiced^45,46^. For example, clinicians recommend inactivated influenza vaccination in pregnancy to reduce the risk of neonatal infection by maternal antibodies^47^. In humans, IgG transfer across the placenta begins in the late first trimester (~17 weeks of gestation) and increases towards term^48,49^. We conducted a passive transfer study to examine the role of vaccine-derived antibodies in protection against ZIKV infection. The results showed that although a single dose of 25 mg vIgG (50 mg kg^−1^ body weight) appeared to be ineffective, two doses of 20 mg vIgG (2 × 40 mg kg^−1^ body weight) substantially reduced viral RNA copies in the placenta and fetuses. The failure of protection by the antibody transfer was surprising, especially given the results from a parallel study using a mouse model, where we observed a dose-dependent defense against ZIKV infection^41^. We consider three possible explanations: (1) a single dose of human IgG may have led to antibody-dependent enhancement of ZIKV, particularly after the 2nd viral injection, rather than neutralization, because a higher vRNA burden was detected in maternal and fetal tissues of marmosets after the single dose human IgG transfer than that of untreated marmosets, hence (2) higher dose antibody transfer prior to each of ZIKV inoculation may be required for protection, and (3) the interaction of human IgG antibodies with Fc receptors in marmosets may be less efficient than their orthologous counterparts, both in terms of affinity and in initiating down-stream non-neutralizing effector functions, which have yet to be explored in marmosets. Nonetheless, these results offer promise and suggest that higher doses of transferred antibody may be necessary to prevent ZIKV transmission in utero, emphasizing the importance of careful dosing and frequency analysis of the antibody-based therapy.

Our studies have emphasized the role of neutralizing antibodies in vaccination against ZIKV. However, T cell-mediated immunity may contribute to the protection against ZIKV infection. Previously, ZPIV has been shown to elicit virus-specific T cell responses in non-pregnant cynomolgus macaques^30^. A comparative study of vaccine-induced T-cell responses before and during pregnancy in marmosets is an important area for further investigation.

In summary, the current study demonstrates the safety of ZPIV vaccination during pregnancy, as indicated by the absence of adverse effects. The vaccine was highly immunogenic, as pregnant marmosets mounted high neutralizing antibody titers comparable to levels observed in non-pregnant marmosets after vaccination, which was reported previously^28^. More importantly, vaccination during pregnancy reduced vertical transmission in pregnant marmosets. Future studies will be required to determine the clinical impact of low vRNA copies detected in the fetal tissues from the vaccinated group because it is unclear whether these low copies translate into infectious particles. Finally, the data reinforce the robustness of the marmoset pregnancy model. This model will allow future studies to determine whether ZPIV vaccination during pregnancy, which we have shown to interfere with virus transmission from mother to fetus, also protects offspring from Zika-associated congenital diseases.

## Methods

### Study design

Seven adult nulliparous females between 2 and 2.5 years old with an average weight of 485 g (±40 g) were enrolled in the study. Marmosets were observed twice daily, and weights were recorded monthly. The male partners were between 3 and 4 years old with an average weight of 430 g (±60 g). The females were individually housed within visual, auditory and olfactory range of the other females and males in the room. Each of female received an unsedated ultrasound examination monthly. Pregnancy can be detected as early as 30 days estimated gestational age. The gestational age of the embryos is estimated using crown-rump length assessed via ultrasound, a method that reliably estimates gestational age in marmosets within +/−3 days^50,51^. Once pregnancy was noted, estimated 30 days of gestational age, the female received a second ultrasound 10–15 days later to confirm the pregnancy. The pregnancy confirmed females received intramuscular injections of 2.5 μg (5 μg kg^−1^ body weight) alum-adjuvanted ZPIV at egd 40 during the 1st trimester and a boost dose at 3 weeks (egd 61) later and two pregnant marmosets remained unvaccinated as controls. Then, 2 weeks (egd 75, the 2nd trimester) after the boost, all marmosets were intramuscularly inoculated with two doses of 2.5 × 10^5^ PFU of ZIKV-BR at egd 75 and 79 during the second trimester, as described previously^27^. To minimize stress-associated negative impact on pregnancy, blood samples were collected at 2 weeks after each of the vaccination and −5, 2, 9, 14 days after the challenge. Urine samples were collected at −1, 1, 3, 7, 10 and 14 days after the challenge. At 14 dpi (edg 89 ± 3), females were sedated with ketamine (15 mg/kg IM) and euthanized by i. v. injection of 0.5–1.0 mL Fatal-Plus (Patterson Veterinary) containing 390 mg/mL pentobarbital sodium at necropsy to yield placenta and fetuses. The placentas were removed from the uterus using sterile technique, the placental discs were separated, and each fetus was removed. The number of fetuses ranged from 2 to 4 per dam. Each fetus was measured and photographed prior to dissection. The head was removed and cut in half along the sagittal plane. One half of the fetal head was weighed and frozen in liquid nitrogen instantly and the other half was placed in a cassette in 10% neutral buffered formalin (NBF, Fisher Scientific). The fetal body was weighed and frozen instantly in liquid nitrogen. All frozen tissues were stored at −80 °C, shipped to Trudeau Institute and processed for RNA isolation. Each disc of the placenta was weighed and photographed. One intact disc was fixed in 10% NBF, the other disc was divided into four equal pieces with two pieces ranging from 0.03 to 0.1 gram each being placed in liquid nitrogen, the other pieces were placed into a cassette and stored in 10% NBF for histology. Maternal spleens and mesenteric lymph nodes were harvested and frozen instantly in liquid nitrogen. Note that viral RNA loads and neutralizing antibody titers from two unvaccinated ZVC marmosets were presented previously^52^ as the unvaccinated ZVC group was shared between two projects conducted simultaneously. As a control, an age-matched and gestational day-comparable unvaccinated, virus-free control dam was included in the study.

For the passive transfer study of human purified polyclonal IgG (Supplementary Fig. 1), three female marmosets between 2 and 2.5 years old were enrolled for the study and cared for the same as the marmosets for the vaccine study as described above. At egd 65, two females were sedated and intravenously infected with a single dose of 25 mg hu-pIG on day 0, egd 65 or one female was injected with two doses of 20 mg at egd 65 and 69. Two hours after antibody transfer, the marmosets were intramuscularly inoculated with 2.5 × 10^5^ PFU of ZIKV-BR twice at egd 65 and 69. At 14 dpi (edg 79), females were sedated with ketamine (15 mg/kg IM) and euthanized for further examination.

### Ethics statement

The research was conducted in compliance with the Animal Welfare Act and other federal statutes and regulations relating to animals and experiments involving animals and adheres to principles stated in the Guide for the Care and Use of Laboratory Animals, NRC Publication, 2011 8^th^ edition.

All marmosets were housed in the AAALAC-accredited animal facility at the Southwest National Primate Research Center (SNPRC), located at Texas Biomedical Research Institute. The general animal care, diet and enrichment for the colony at SNPRC has been previously described^53^. The local IACUC and Biohazard Committee reviewed and approved all marmoset study protocols prior to the initiation at Texas Biomedical Research Institute (TBRI), and the studies were performed accordingly.

All postmortem samples were shipped to Trudeau Institute for further analysis, and serum samples were shipped to Walter Reed Army Institute of Research (WRAIR) for examination of virus neutralizing activity.

### Zika virus

A low passage working stock (passage 2) of the ZIKV-Brazil SPH 2015 strain was originally obtained from Dr. Lark Coffey (Univ. of California-Davis) and used for infecting marmosets. Vero cells were purchased from American Type Culture Collection (ATCC, CCL-81) and used for virus neutralization assay.

### Zika purified inactivated virus (ZPIV) vaccine

ZPIV is a purified, formalin inactivated Zika virus vaccine developed by WRAIR as described previously^29^. Briefly, the Puerto Rico strain, PRVABC59 of ZIKV was initially obtained from the Centers for Disease Control and Prevention (Fort Collins, CO, USA) and propagated in a qualified Vero cell line. After purification using chromatography-column and inactivation, the virus was absorbed in a 1:1 ratio with 1 mg/mL alum (Alhydrogel, Brentagg Biosector, Frederikssund, Denmark). Alum-absorbed ZPIV was prepared at the concentration of 10 μg/mL.

### RNA isolation

Each of the frozen tissue samples were resuspended in RLT buffer (Qiagen) containing β-mercaptoethanol (β-ME) at the concentration of 100 mg/mL and homogenized with stainless steel beads using a TissueLyzer II instrument (Qiagen). For the lipid-rich brains, Trizol (Thermo-Fisher) was added to prepare homogenates followed by addition of chloroform. Then, RNA extractions from aqueous phase were processed with the RNeasy mini kit (Qiagen) according to the manufacturer’s instructions^54^. RNA pellets were resuspended in 60 µL of RNase-free distilled water, quantified using a NanoDrop 2000 (NanoDrop Technologies, Wilmington, DE) and stored frozen at −70 °C.

### One-step real-time quantitative reverse transcription-polymerase chain reaction (qRT-PCR)

One-step RT-qPCR was performed on a 7500 Fast real-time PCR system (Applied Biosystems) to quantify ZIKV RNA using primers and probe sequences as described previously^55^, which are 5′-CCGCTGCCCAACACAAG-3′, reverse 5′-CCACTAACGTTCTTTTGCAGACAT-3′, probe 5′-/56-FAM/AGCCTACCT/ZEN/TGACAAGCAGTCAGACACTCAA/3IABkFQ/-3′ (Integrated DNA Technologies). The PCR conditions were optimized using 1 µg total RNA in a 20 µL reaction cocktail containing TaqMan Fast Virus 1-step Master Mix (Applied Biosystems), 5 pM primers, and 20 pM probe (IDT, Coralville, IA). Reverse transcription was performed at 50 °C for 15 min, 95 °C for 2 min immediately followed by 45 cycles of 95 °C for 15 s, 60 °C for 30 s. for qPCR, and the data was analyzed 7500 Fast software (version 1.4). Viral RNA levels were interpolated against standard curves prepared by diluting RNA from uninfected Vero cells spiked with a known copy number of ZIKV genomic RNA (NR-50244) obtained from BEI Resources (Manassas, VA). As described previously^28^, we defined the limit of detection as the cycle of threshold (Ct) equal to 37 and the limit of quantitation as Ct value ≤ 35 with 100% positivity of PCR runs of the standard control. We examined serial dilutions of ZIKV-BR stock and found the limit of quantification (LLOQ) for viral RNA to be 0.001 FFU/mL, corresponding to a mean Ct value of 35.7 in triplicated reactions. Based on Ct values corresponding to viral RNA copy numbers in our assay conditions, 1 FFU is estimated to be equivalent to approximately 3–5 × 10^3^ copies of vRNA per mL or per gram tissue.

### Microneutralization (MN_50_) assay

ZIKV neutralizing antibody titers were determined using a high throughput microneutralizing antibody assay at WRAIR as described previously^28^. Briefly, all serum samples were heat incubated at 56 °C for 30 min and diluted in PBS at 1:10 and tested 8 serial dilutions per sample. The serum dilutions were mixed with 100 PFU of ZIKV PRVABC59 per well. Following incubation at 35 °C for 2 h, the mixtures were added to 96-well plates containing Vero cell monolayers in triplicate wells and the plates were incubated for 4 days. Then, following the washing, fixing, and blocking steps, the plates were incubated with pan-flavivirus monoclonal antibody, clone 6B6-C1 (a gift from J. T. Roehrig, U.S. Centers for Disease Control and Prevention) conjugated with HRP for 2 h. The plates were then washed and incubated with TMB substrate for 50 min at RT. The enzymatic reaction was stopped by adding 1:25 phosphoric acid, and the absorbance was measured optical density (OD) at 450 nm. Fifty percent micro-neutralization (MN_50_) titers were determined as the reciprocal serum dilution corresponding to that the wells reducing OD values by 50% when compared with that of the wells containing 100 PFU of virus alone.

### Histology and evaluation for pathology in the placenta of marmosets

Tissues were fixed with 10% neutral buffered formalin (Fisher Scientific) and processed into paraffin blocks. Sections were cut at 5 μm thickness and mounted on charged glass slides (Fisher Scientific). The slides were stained with hematoxylin and eosin following a previously described protocol^52^. For unbiased evaluation, paraffin-embedded tissue blocks were prepared at 3-8 different locations per placenta including near the umbilical cord attachment and other peripheral locations. Hematoxylin and eosin slides of marmoset placentas were evaluated by a board-certified veterinary pathologist. Histopathologic features were recorded for each of the placental regions: decidua, chorionic villi, intervillous space and amnion. Severity of inflammation and necrosis was scored on a scale of 0–3, which defined as 0 = normal, 1 = minimal, 2 = mild and 3 = moderate per placental region. Then, the final semiquantitative score of individual placentas was calculated by totaling the inflammation and necrosis scores for each section of placenta evaluated. The numbers of the sections examined per group are VFC (*n* = 3), ZVC (*n* = 9), and ZPIV (*n* = 26). Fibrin deposition in the placenta was quantified using HALO Image Analysis tissue classifier and HALO Image Analysis Platform Software V.3.5.3566 (Indica Labs, Inc., Albuquerque, NM). Slides of placental tissues, stained with hematoxylin and eosin were scanned using a ZEISS Slide Scanner Axio Scan.Z1 and images were obtained with ZEN 3.4 Digital Imaging for Light Microscopy (Zen lite blue edition). Image panels were prepared using Adobe photoshop.

### Preparation of human immunoglobulin G from unvaccinated normal donors and ZPIV vaccinees

Flavivirus naïve individuals received three ZPIV immunizations. Based on high ZIKV neutralizing antibody titers at days 252 and 308, one- and three-months post third ZPIV dose, respectively, serum samples were selected from 19 individual donors^32^. The individual samples were heat-inactivated, centrifuged at 20,000 × *g* for 5 min and pooled. Commercially available normal human serum (Sigma, H4522) was purchased and was tested for the lack of ZIKV neutralization activity. Human polyclonal IgG antibodies were purified using Protein G Sepharose (Cytiva, 17061801) columns as described previously^41^. After extensive washes in 1× PBS pH 7.4, bound IgG were eluted with 0.5 M acetic acid, pH 3.0, quickly neutralized with 3 M Tris, concentrated and buffer exchanged to 1× PBS pH7.4 and sterile filtered. Concentrated material was quantified using Nanodrop spectrophotometer. Purity and identity were confirmed by western blot SDS-PAGE gel and biological function was characterized^41^.

### Statistical analysis

All data were analyzed using GraphPad Prism software v 9.2.0 (San Diego, CA). Viral RNA levels were analyzed using unpaired non-parametric Mann–Whitney test between groups. The comparison of antibody response between groups and different times was analyzed using ANOVA test. Histopathology data of the individual animals was compared with that of virus-free control dam using one-way ANOVA test.

### Reporting summary

Further information on research design is available in the Nature Research Reporting Summary linked to this article.

### Supplementary information

Supplementary information

dataset 1

dataset 2

REPORTING SUMMARY

## Data Availability

The data that support the findings of this study are available at 10.6084/m9.figshare.25006826 or contact the corresponding authors with a written request.
