# Supplementary material for: Protective efficacy of a Zika purified inactivated virus vaccine candidate during pregnancy in marmosets
Source: NPJ Vaccines. 2024 Feb 17;9:35. doi: 10.1038/s41541-024-00824-0 (PMC10874403; doi:10.1038/s41541-024-00824-0)
Supplement: Supplementary file 1 — Supplementary information [file 41541_2024_824_MOESM1_ESM.docx]

**Protective efficacy of Zika purified inactivated virus vaccination during pregnancy in marmosets**

In-Jeong Kim^1*^, Olga Gonzalez^2^, Michael P. Tighe^1^, Paula A. Lanthier^1^, Madeline J. Clark^1^, Kelsey L. Travis^1^, Timothy C. Low-Beer^1^, Kathleen G. Lanzer^1^, Derek T. Bernacki^1^, Frank M. Szaba^1^, Rafael A. De La Barrera^3^, Vincent Dussupt^4, 5, 6^, Letzibeth Mendez-Rivera^4, 5, 6^, Shelly J. Krebs^4, 5, 6^, Corinna N. Ross^2^, Stephanie D. Mdaki^2†^, Kathleen M. Brasky^2^, Donna Layne-Colon^2^, Suzette D. Tardif ^2^, Stephen J. Thomas^8^, Kayvon Modjarrad^4^, Marcia A. Blackman^1^, and Jean L. Patterson^2*^

**Supplementary Information**

**Table of Contents**

**Supplementary Figure 1**

**Supplementary Figure 2**

**Supplementary Table 1**

**Supplementary notes**

**Supplementary References**

**
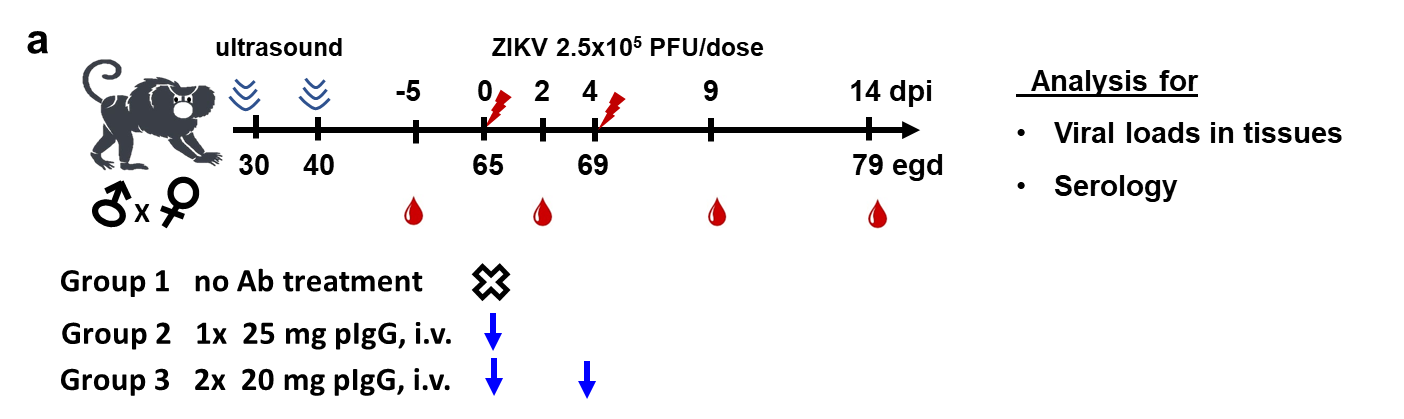
**

**Supplementary Figure 1. Protective effect of antibody transfer against perinatal ZIKV challenge in marmosets**

Two hours prior to ZIKV infection, pregnant marmosets were injected with 0 mg (n=2), a single dose of 25 mg (n=2) and two doses of 20 mg (n=1) at days 0 and 4, estimated gestational day 65 and 69. All marmosets were i.m. injected with 2.5 x10^5^ PFU of ZIKV-BR at egd 65 and 69 and examined at 14 dpi, egd 79 (a). Viral RNA copies were quantified in maternal mesenteric lymph nodes (b), placenta (c), fetal heads (d) and fetal body (e) using qRT-PCR. All individual samples were tested in duplicate reactions. Symbols represent individual samples, each indicating the mean of duplicated testing. For fetal tissues (d and e), the number of fetuses examined are 5, 5, and 3 per 0 mg, 1x, 25 mg, and 2x, 20 mg treatment, respectively. No statistical difference in viral loads in fetal tissues between untreated and treated groups was detected in d and e (*P* > 0.05).

**Supplementary Figure 2.** **Antibody response in pregnant marmosets after perinatal ZIKV challenge**

Serum samples prepared at indicated days after antibody transfer were examined for neutralizing activity using a microneutralization assay (a), and ZIKV-specific IgG titers (b), and marmoset IgM titers in the recipients at 14 dpi (c) using Luminex assay. Dotted lines indicate the highest serum dilution 1:5 or 1:10 examined. Based on background signal, MFI values equal to or higher than 120 or 150 were used to determine end-point reciprocal serum titers for IgM and IgG specific for ZIKV, respectively. Due to the limited samples size, statistical analysis is not feasible.

**Supplementary Table 1. Virus neutralizing antibody titers (log10 MN_50_ titer) after ZPIV vaccination before or during pregnancy in marmosets**

| Sample ID | Week 2 post-boost | | Day 9 post-challenge | | Wilcoxon test |
| --- | --- | --- | --- | --- | --- |
| Vaccination prior to pregnancy | | | | | |
| V1 | 3.677 | | 3.863 | |  |
| V2 | N.T. | | 3.803 | |  |
| V3 | 3.614 | | 3.702 | |  |
| V4 | 3.863 | | 4.328 | |  |
| **GMT** | **3.716** | | **3.917** | | ***P* = 0.250** |
| lower C.I. | 3.411 | | 3.511 | |  |
| upper C.I. | 4.050 | | 4.37 | |  |
| Vaccination during pregnancy | | | | | |
| ZPIV1 | 3.268 | | 3.863 | |  |
| ZPIV2 | 3.010 | | 3.863 | |  |
| ZPIV3 | 3.418 | | 4.357 | |  |
| ZPIV4 | 3.238 | | 4.406 | |  |
| **GMT** | **3.230** | | **4.114** | | ***P* = 0.125** |
| lower C.I. | 2.971 | | 3.664 | |  |
| upper C.I. | 3.513 | | 4.620 | |  |
| Mann-Whitney test | ***P* = 0.0571** | ***P* = 0.2000** | |  | |

Marmosets (V1-4) were intramuscularly immunized with 2.5 μg ZPIV at week 0 and 4 prior to pregnancy from the previously published work^28^. Marmosets (ZPIV 1-4) were intramuscularly immunized ZPIV at week 0 and 3 during pregnancy (egd 40-45 and 61-66, respectively). Virus neutralizing antibody titers in serum samples were determined at 2 weeks post-boost and day 9 post-challenge using microneutralizing assay. Data are presented as log10 MN_50_ titers and analyzed to detect the difference between antibody titers between groups vaccinated prior to and during pregnancy using the unpaired Mann-Witney test. The data are also analyzed for the difference in antibody titers at different times within the group using the paired rank Wilcoxon test. N.T., not tested; ZPIV, zika purified inactivated virus; C.I., confident interval; GMT, geometric mean.

**Supplementary notes**

**Comparable virus-neutralizing antibody response between marmosets with pre-pregnancy vaccination and marmosets with vaccination during pregnancy**

To directly address the comparability of the neutralizing antibody titer elicited by ZPIV vaccination during pregnancy in marmosets, we compared to those from pre-pregnancy vaccination from our previous published work^28^ (Supplementary Table 1). In the pre-pregnancy vaccine study, marmosets became pregnant at different times after receiving a boost dose of ZPIV several months apart. To be consistent with the current study condition, we compared log10 MN_50_ titers at 2 weeks post-boost and day 9 post-challenge, the most relevant timepoints after ZPIV boost between and after ZIKV challenge between pre- and during pregnancy vaccination regimens. The data indicate no significant difference in virus neutralizing antibody titers between the animals (V1-V4) that ZPIV was given before or during pregnancy (ZPIV1-4), supporting the statement that vaccination during pregnancy induces comparable virus-neutralizing antibody response. Despite the comparable neutralizing antibody titers, it is unexpected that the ZPIV group showed detectable viral RNA in maternal serum tissue samples. The current results show that ZPIV vaccination in pregnant marmosets elicits quantitatively comparable virus neutralizing antibody titers. However, the results may imply that pregnancy-associated immunosuppression affects the quality of antibody response. A comparative study of the effector function of the antibody response indued by vaccination, either before or during pregnancy, is of interest for future investigations.

**Passive antibody transfer fails to protect marmosets against ZIKV infection**

Maternal antibodies transferred through placenta or breast-feeding can protect newborns who are at high risk for ZIKV infection and developing CZS because their immunity is not yet fully developed^1,2^. We examined whether passive transfer of hu-pIgG into pregnant marmosets was protective against ZIKV infection. Based on the results of the mouse study, the minimum dose required for complete protection is 1 mg dose, equivalent to 37 mg kg^-1^ body weight. Two pregnant marmosets (pIgG1 and pIgG2) received a single dose of 25 mg (eq. 50 mg kg^-1^ body weight) (Supplementary Figure 1a). Despite different treatments, viral RNA copies in maternal lymph nodes were similar with the untreated controls (ZVC1 and ZVC2), suggesting that the virus may shed in lymph nodes (Supplementary Figure 1b). Viral RNA copies in the placentas and fetal tissues (n=3) of the marmoset, pIgG3, that received two doses of 20 mg were reduced compared with untreated controls but not in the fetal tissues (n=5) of two marmosets that received a single dose of 25 mg (Supplementary Figure 1c-e). Due to the limited sample size, statistical analysis is not warranted.

**Antibody response in the recipients after passive transfer in marmosets**

Neutralizing antibody titers in pIgG 3 (log10 MN_50_, 1.9) remained relatively low than titers of pIgG1 and pIgG2, and the control ZVC2 (log10 MN_50_ > 3.0) by 9 dpi. However, neutralizing antibody titers in all marmosets became comparable by 14 dpi (Supplementary Figure 2a) regardless of different treatments, suggesting delayed host antibody response by the passive antibody transfer.

Because of the cross-species reactivity, it was not possible to discern human IgG (adoptively transferred antibody) from marmoset IgG (recipient). We could partially discern the donor and host antibodies based on the time elapsed after antibody transfer and ZIKV infection. Antibodies detected before 5 dpi were likely primarily donor IgGs, since it is too early for the recipient to generate detectable primary antibody response, whereas IgG detected after 9 dpi would be predominantly recipient-derived antibody generated by active immunity. Indeed, relatively high IgG levels were detected at day 0, 2 hours after antibody administration prior to ZIKV infection, in pIgG1 and pIgG2 marmosets than pIgG3 marmoset (Supplementary Figure 2b). However, at 14 dpi, ZIKV-specific IgM titers in the recipients, pIgG 1-3, became comparable with the titers in control ZVC marmosets (Supplementary Figure 2c). The results suggest that the passive antibody transfer failed to prevent ZIKV infection, and the virus elicited a primary antibody response in the recipients. At least, the recipient received two doses of antibody transfer showed IgG titer close to the limit of detection. Together with reduced viral burden in fetal tissues, these results suggest that higher doses or more frequent administration of the antibody are required to confer protection against ZIKV infection.

**Supplementary References**

1 Mavigner, M. *et al.* Postnatal Zika virus infection is associated with persistent abnormalities in brain structure, function, and behavior in infant macaques. *Sci Transl Med* **10**, doi:10.1126/scitranslmed.aao6975 (2018).

2 Raper, J. *et al.* Long-term alterations in brain and behavior after postnatal Zika virus infection in infant macaques. *Nat Commun* **11**, 2534, doi:10.1038/s41467-020-16320-7 (2020).
