# Supplementary material for: Protective efficacy of a Zika purified inactivated virus vaccine candidate during pregnancy in marmosets
Source: NPJ Vaccines. 2024 Feb 17;9:35. doi: 10.1038/s41541-024-00824-0 (PMC10874403; doi:10.1038/s41541-024-00824-0)
Supplement: Supplementary file 4 — REPORTING SUMMARY [file 41541_2024_824_MOESM4_ESM.pdf]

nature portfolio

Corresponding author(s): In-Jeong Kim
Jean L. Patterson

Last updated by author(s): Dec 19, 2023

Reporting Summary

Nature Portfolio wishes to improve the reproducibility of the work that we publish. This form provides structure for consistency and transparency in reporting. For further information on Nature Portfolio policies, see our Editorial Policies and the Editorial Policy Checklist.

Statistics

For all statistical analyses, confirm that the following items are present in the figure legend, table legend, main text, or Methods section.

n/a	Confirmed	
☐	☒	The exact sample size (*n*) for each experimental group/condition, given as a discrete number and unit of measurement
☐	☒	A statement on whether measurements were taken from distinct samples or whether the same sample was measured repeatedly
☐	☒	The statistical test(s) used AND whether they are one- or two-sided<br>*Only common tests should be described solely by name; describe more complex techniques in the Methods section.*
☐	☒	A description of all covariates tested
☒	☐	A description of any assumptions or corrections, such as tests of normality and adjustment for multiple comparisons
☐	☒	A full description of the statistical parameters including central tendency (e.g. means) or other basic estimates (e.g. regression coefficient) AND variation (e.g. standard deviation) or associated estimates of uncertainty (e.g. confidence intervals)
☐	☒	For null hypothesis testing, the test statistic (e.g. *F*, *t*, *r*) with confidence intervals, effect sizes, degrees of freedom and *P* value noted<br>*Give P values as exact values whenever suitable.*
☒	☐	For Bayesian analysis, information on the choice of priors and Markov chain Monte Carlo settings
☒	☐	For hierarchical and complex designs, identification of the appropriate level for tests and full reporting of outcomes
☒	☐	Estimates of effect sizes (e.g. Cohen's *d*, Pearson's *r*), indicating how they were calculated

Our web collection on statistics for biologists contains articles on many of the points above.

Software and code

Policy information about availability of computer code

Data collection	The following softwares were used for collect raw data for RealTime PCR, and optical density:<br>Applied System 7500 Fast System software V1.4.0,<br>SoftMax Pro V 7.1.2
Data analysis	GraphPad Prism Software v.9.2

For manuscripts utilizing custom algorithms or software that are central to the research but not yet described in published literature, software must be made available to editors and reviewers. We strongly encourage code deposition in a community repository (e.g. GitHub). See the Nature Portfolio guidelines for submitting code & software for further information.

Data

Policy information about availability of data

All manuscripts must include a data availability statement. This statement should provide the following information, where applicable:

- Accession codes, unique identifiers, or web links for publicly available datasets
- A description of any restrictions on data availability
- For clinical datasets or third party data, please ensure that the statement adheres to our policy

All data presented in this publication are available upon request to corresponding authors with the permission by Walter-Reed Army Institute of Research.

Research involving human participants, their data, or biological material

Policy information about studies with human participants or human data. See also policy information about sex, gender (identity/presentation), and sexual orientation and race, ethnicity and racism.

Reporting on sex and gender	Serum samples from 10 females and 10 males who were flavivirus naive at the time of the study (clinical trial NCT02963909) enrollment according to Koren et al., 2023 (https://doi.org/10.1016/S1473-3099(23)00192-5)
Reporting on race, ethnicity, or other socially relevant groupings	According to the report (Table 1) by Koren et al., 2023 (https://doi.org/10.1016/S1473-3099(23)00192-5), eligible participants were adults aged 18–49 years, 3 participants are Hispanic or Latino and 17 participants are not Hispanic or Latino. Of the 17 not Hispanic or Latinos, 10 are White, 7 are black/African American and 1 are multiracial. Pregnant or breastfeeding women were excluded. All study participants were required to be generally physically healthy and without any clinically significant medical conditions including any immunodeficiency (or use of immunosuppressive medication); history of malignancy; acute or chronic neurological conditions; diabetes type 1 or type 2; thyroid disease; major psychiatric illness; autoimmune disease; hypercholesterolaemia; chronic hepatitis or cirrhosis; or chronic pulmonary, renal, or cardiac disease.
Population characteristics	N.A.
Recruitment	Participants were recruited through online, printed, and emailed advertisements. Participants (n=25) were recruited sequentially into a flavivirus naive group
Ethics oversight	Serum samples from human subjects were from the RV478 study, "A Phase 1, First-in-human, Double-blinded, Randomized, Placebo-controlled Trial of a Zika Virus Purified Inactivated Vaccine (ZPIV) with alum adjuvant in Healthy Flavivirus-naive and Flavivirus-Primed Subjects". The WRAIR Institutional Review Board (IRB) approved the protocol prior to the study initiation. Written informed consent was obtained from all participants before screening. The investigators have adhered to the policies for protection of human participants as prescribed in AR 70–25. The trial is registered at ClinicalTrial.gov number: NCT02963909.

Note that full information on the approval of the study protocol must also be provided in the manuscript.

Field-specific reporting

Please select the one below that is the best fit for your research. If you are not sure, read the appropriate sections before making your selection.

☒ Life sciences ☐ Behavioural & social sciences ☐ Ecological, evolutionary & environmental sciences

For a reference copy of the document with all sections, see nature.com/documents/nr-reporting-summary-flat.pdf

Life sciences study design

All studies must disclose on these points even when the disclosure is negative.

Sample size	A total of 7 marmosets were included in the study:<br>1, virus-free marmoset as an age- and gestational day-comparable control<br>2, unvaccinated, ZIKV infected animals,<br>4, vaccinated, ZIKV infected animals
Data exclusions	No data were excluded for data analysis.
Replication	No repeat study was included. Individual samples were examined in duplicates or triplicates whenever applicable.
Randomization	Based on age and body weight, marmosets were initially randomly assigned for the study. Then, based on the time of pregnancy confirmation, marmosets were vaccinated during pregnancy. All animals were examined during comparable gestational days 89-94 or at 14 days after ZIKV infection.
Blinding	Not blinded due to the small sample sizes of the experimental groups.

Reporting for specific materials, systems and methods

We require information from authors about some types of materials, experimental systems and methods used in many studies. Here, indicate whether each material, system or method listed is relevant to your study. If you are not sure if a list item applies to your research, read the appropriate section before selecting a response.

Materials & experimental systems

n/a	Involved in the study	
☐	☒	Antibodies
☐	☒	Eukaryotic cell lines
☒	☐	Palaeontology and archaeology
☐	☒	Animals and other organisms
☒	☐	Clinical data
☐	☒	Dual use research of concern
☒	☐	Plants

Methods

n/a	Involved in the study	
☒	☐	ChIP-seq
☒	☐	Flow cytometry
☒	☐	MRI-based neuroimaging

Antibodies

Antibodies used	Purified human IgG antibodies were serum samples obtained from human subjects. Serum samples of the human subjects were from the RV478 study, "A Phase 1, First-in-human, Double-blinded, Randomized, Placebo-controlled Trial of a Zika Virus Purified Inactivated Vaccine (ZPIV) with alum-adjuvant in Healthy Flavivirus-naive and Flavivirus-Primed Subjects". The WRAIR Institutional Review Board (IRB) approved the protocol prior to the study initiation. The trial is registered at ClinicalTrial.gov number: NCT02963909
Validation	Information of purified human IgG antibodies is indicated in the Supplementary information of the manuscript.

Eukaryotic cell lines

Policy information about cell lines and Sex and Gender in Research

Cell line source(s)	The green monkey kidney epithelial cells, Vero cells (CCL-81) were purchased from American Tissue and Cell Culture (ATCC).
Authentication	Certificate of Analysis of the Vero cells is available from ATCC.
Mycoplasma contamination	Vero cell culture supernatant was tested for Mycoplasma pulmonis and other Mycoplasma species, prior to virus-replication in vitro and the result confirmed to be free of the Mycoplasma.(IMPACT test Case # 24231-2016 by IDEXX BioResearch)
Commonly misidentified lines (See ICLAC register)	N.A.

Animals and other research organisms

Policy information about studies involving animals; ARRIVE guidelines recommended for reporting animal research, and Sex and Gender in Research

Laboratory animals	Common marmosets (Callithrix jacchus) were bred and maintained in the Southwestern National Primate Research Center within the Texas Biomedical Research Institute.
Wild animals	N.A.
Reporting on sex	Pregnant female marmosets and their fetuses were used for the study
Field-collected samples	N.A.
Ethics oversight	All marmosets were housed in the AAALAC-accredited animal facility at the Southwest National Primate Research Center (SNPRC), located at Texas Biomedical Research Institute. The animal care, diet and treatment were managed at SNPRC. IACUC and Biohazard Committee reviewed and approved all marmoset study protocols, 1510CJ and 1610 CJ, prior to the initiation at Texas Biomedical Research Institute (TBRI), and the studies were performed accordingly. The marmoset studies were also approved by by the Animal Care and Use Review Office (ACURO) at the Department of Defense prior to initiation of the study.

Note that full information on the approval of the study protocol must also be provided in the manuscript.

Plants

Seed stocks	N.A.
Novel plant genotypes	N.A.
Authentication	N.A.
